# The Effectiveness of Transforaminal Versus Caudal Routes for Epidural Steroid Injections in Managing Lumbosacral Radicular Pain: A Systematic Review and Meta-Analysis

**DOI:** 10.1097/MD.0000000000003373

**Published:** 2016-05-06

**Authors:** Jun Liu, Hengxing Zhou, Lu Lu, Xueying Li, Jun Jia, Zhongju Shi, Xue Yao, Qiuli Wu, Shiqing Feng

**Affiliations:** From the Department of Orthopedics (JL, HZ, LL, JJ, ZS, XY, QW, SF), Tianjin Medical University General Hospital, No. 154 Anshan Road; Key Laboratory of Immuno Microenvironment and Disease of the Educational Ministry of China (XL), Department of Immunology, Tianjin Medical University, No. 22 Qixiangtai Road, Heping District; and Department of Orthopedic Trauma (JJ), Tianjin Hospital, No. 406 Jiefangnan Road, Hexi District, Tianjin, PR China.

## Abstract

Epidural steroid injection (ESI) is one of the most commonly used treatments for radiculopathy. Previous studies have described the effectiveness of ESI in the management of radiculopathy. However, controversy exists regarding the route that is most beneficial and effective with respect to the administration of epidural steroids, as both transforaminal (TF) and caudal (C) routes are commonly used.

This analysis reviewed studies comparing the effectiveness of TF-ESIs with that of C-ESIs in the treatment of radiculopathy as a means of providing pain relief and improving functionality. This meta-analysis was performed to guide clinical decision-making.

The study was a systematic review of comparative studies.

A systematic literature search was performed using the PubMed, EMBASE, and Cochrane Library databases for trials written in English. The randomized trials and observational studies that met our inclusion criteria were subsequently included. Two reviewers, respectively, extracted data and estimated the risk of bias. All statistical analyses were performed using Review Manager 5.3.

Six prospective and 2 retrospective studies involving 664 patients were included. Statistical analysis was performed utilizing only the 6 prospective studies. Although slight pain and functional improvements were noted in the TF-ESI groups compared with the C-ESI groups, these improvements were neither clinically nor statistically significant.

The limitations of this meta-analysis resulted primarily from the weaknesses of the comparative studies and the relative paucity of patients included in each study.

Both the TF and C approaches are effective in reducing pain and improving functional scores, and they demonstrated similar efficacies in the management of lumbosacral radicular pain.

## INTRODUCTION

Radicular pain secondary to spinal disease is one of the most challenging medical problems faced by clinicians with respect to therapeutic management. The chemical mediators originating from either a ruptured disc or from neighboring structures, and the mechanical deformation caused by either a herniated disc or excessive tissue proliferation, which results in both nerve root compression and nerve irritation, represent 2 crucial factors that provoke inflammatory responses and increase sensory neuron susceptibility, resulting in radicular pain.^1–3^

Lumbar epidural steroid injection (LESI) was first suggested as a conservative treatment for radicular pain in 1952 by Robecchi and Capra,^4^ and it has since become one of the most commonly utilized conservative interventions for radiculopathy.^5^ Steroids are used to reduce inflammation in the epidural space.^6–10^ LESI is performed via a transforaminal (TF), caudal (C), or interlaminar (IL) approach in the lumbar spine; these approaches offer different advantages and disadvantages, which may result in different outcomes.^11–14^ The TF approach is perhaps the most favored because the injection site is adjacent to the nerve root, and only a small volume of medication is required for injection.^15^ The C route is both the easiest and the safest route and also seems to provide the most favorable analgesic effects. However, this approach requires relatively large volumes of medication and is less specific to the site of pathology.^16^ Previous studies have described the effectiveness of these methods in the management of radiculopathy.^17–22^ The utilization of lumbosacral TF-ESIs has increased annually at a rate of 20.3%, which is markedly higher than the 2% annual increases noted with respect to lumbosacral C-ESI and IL injection based on analysis of data from the Centers for Medicare and Medicaid Services (CMS) collected between 2000 and 2011.^23^

Transforaminal ESI seems to be more effective at reducing pain, improving functionality, and preventing spinal surgery, based on the data reported in previous studies and systematic reviews.^18,24–27^ Some relevant research has already been conducted comparing the effectiveness of the TF versus IL route and the effectiveness of the 3 routes, but no comparison of the TF versus C route has been performed. However, it remains debatable whether TF or C approaches should be utilized in clinical practice, and no definitive standards pertaining to LESI exist. It is therefore necessary to compare the clinical efficacies of different procedures to generate data that can be used to formulate clinical guidelines. Our goal was to systematically review, grade, and perform meta-analysis of existing comparative studies. In this review, we compared the effectiveness of TF-ESIs and C-ESIs with respect to pain and functional improvements in the treatment of radiculopathy.

## METHODS

### Ethics

Ethical approval of this study was not necessary, as systematic review and meta-analyses do not involve patients.

### Study Design

The standards set by the Preferred Reporting Items for Systematic Reviews and Meta-Analyses (PRISMA) guidelines were used to construct this systematic review. The 27-item checklist and 4-phase flow diagram of PRISMA were both consulted.

### Literature Search

The PubMed, EMBASE, and Cochrane Library databases were searched from January 1966 through June 2015 to identify studies comparing TF-ESIs with C-ESIs for the treatment of lumbosacral radicular pain. The search terms included “Transforaminal Epidural Steroid Injection,” “Caudal Epidural Steroid Injection,” “Efficacy of Transforaminal Epidural Steroid Injection,” “Efficacy of Caudal Epidural Steroid Injection,” “Transforaminal versus Caudal Epidural Steroid Injection,” “Efficacy of Transforaminal versus Caudal Epidural Steroid Injection,” and “Selective Nerve Root Block versus Caudal Epidural Steroid Injection.”

References from each article directly comparing the 2 approaches, in addition to review articles discussing the efficacies of the 2 approaches, were cross-referenced to identify additional relevant studies.

### Inclusion and Exclusion Criteria

For inclusion in the systematic review, the articles were required to meet the following eligibility criteria: patients (>18 years) suffering from lumbosacral radicular pain; papers reporting the results of clinical studies evaluating TF-ESIs and C-ESIs; patients followed for a minimum of 2 weeks; and papers published in English before June 2015. Randomized controlled trials (RCTs) were identified as the primary studies for analysis. For inclusion in statistical analysis, the patients in a particular study must have been randomized to either TF or C groups. Studies were excluded from the analysis if they did not include standardized pain scores within specific follow-up periods or perform statistical analyses of their results. The inclusion criteria for each study are listed in Table 1.

**TABLE 1:**
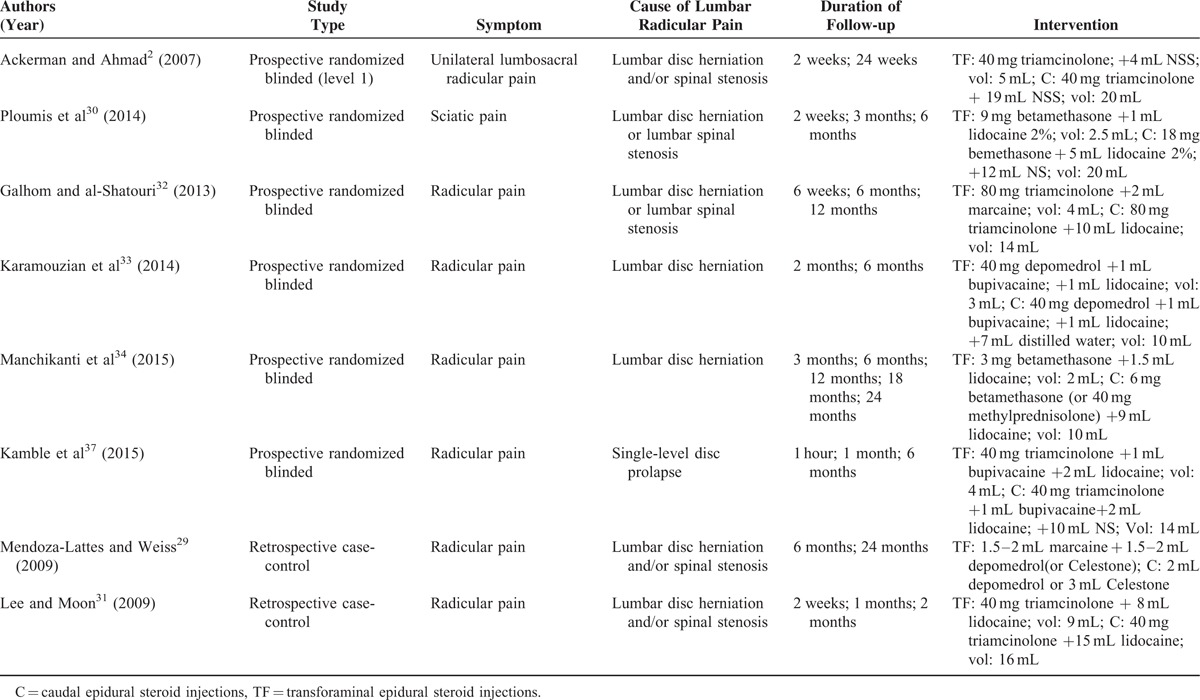
Summary of Study Criteria (Prospective Studies and Retrospective Studies)

### Risk of Bias

The included RCTs were evaluated for the risk of bias, which included assessments of adequate sequence generation, allocation of concealment, blinding, incomplete outcome data, and freedom from other biases, using the Cochrane Risk of Bias Tool. The judgment of each entry involved assessing the risk of bias as “low risk,” “high risk,” or “unclear risk,” indicating either a lack of information or uncertainty over the potential for bias. Two reviewers independently assessed each RCT, and any disagreements were resolved by discussion and consensus.

### Data Extraction

The data were extracted independently by 2 authors (LL and HZ). Any disagreements were resolved via discussion among the 3 reviewers (JL, LL, and HZ). The characteristics of each study were extracted, including the last name of the first author, publication year, study design, number of patients, mean age, baseline pain and functional scores, duration of symptoms, injection level (Table 2), and pain and functional improvement (Table 3).

**TABLE 2:**
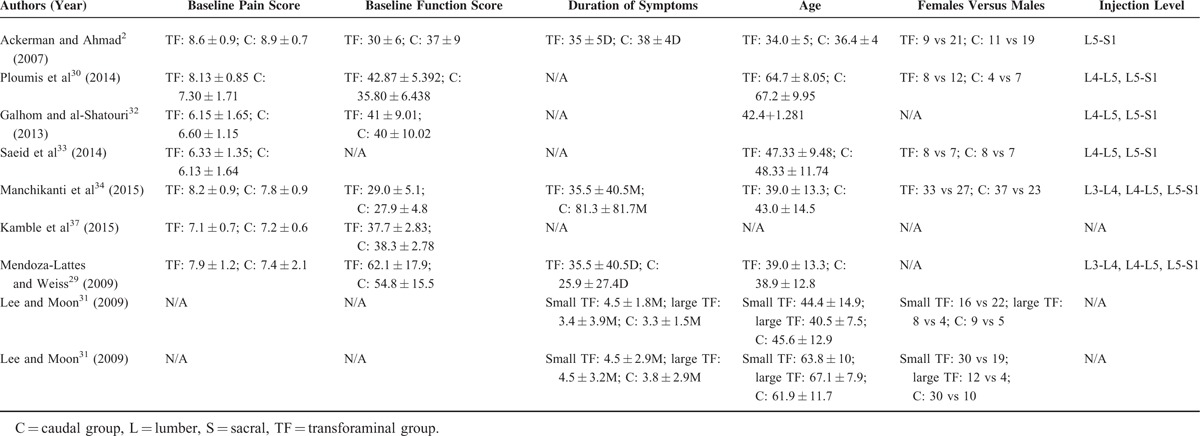
Summary of Baseline Information of the Prospective Studies and Retrospective Studies

**TABLE 3:**
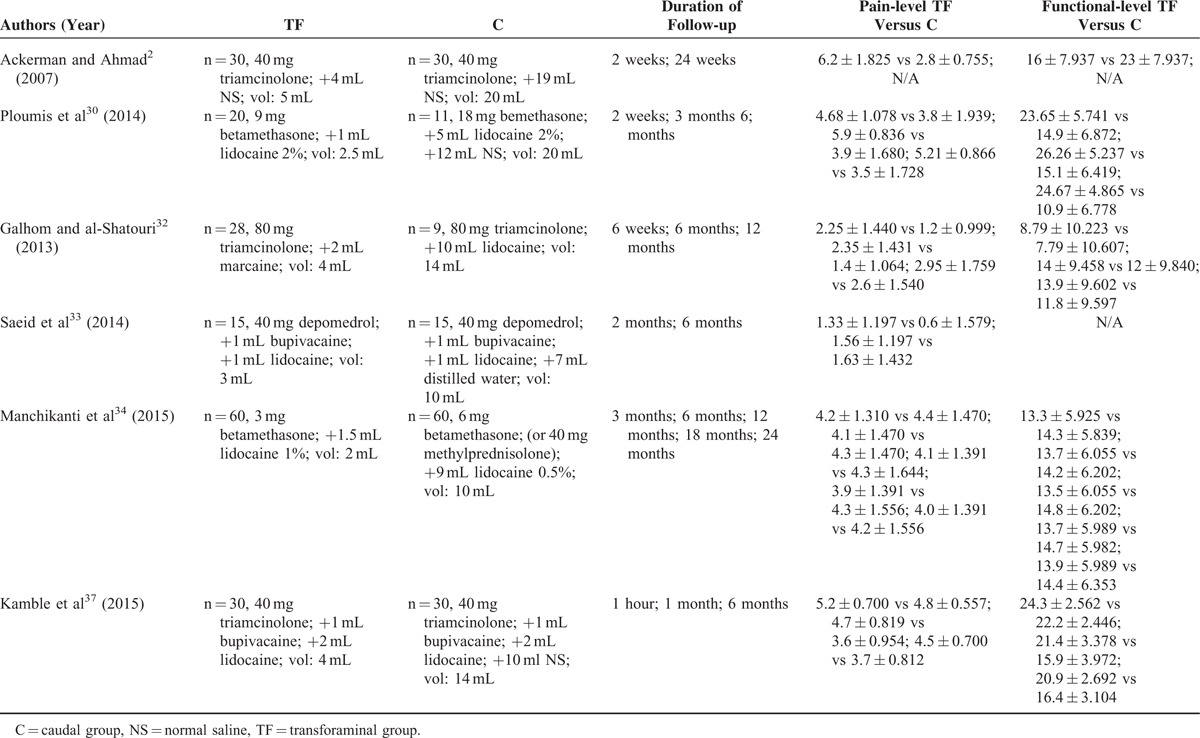
Summary of Head-to-head Studies Comparing TF Versus C

### Outcome Measures

The “degree of pain relief” (visual or verbal analog pain score [VAS]; numerical pain rating scale [NRS]) was the primary outcome measure of the effectiveness of the ESIs. The secondary outcome measure was functional improvement (Oswestry Disability Index [ODI]).

### Statistical Analysis

**FIGURE 1:**
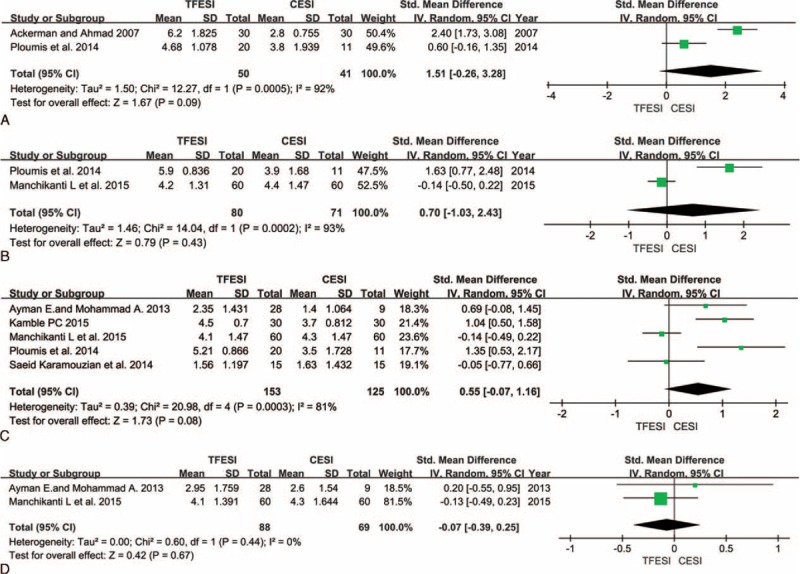
Comparison of TF versus C with respect to pain level after epidural steroid injections at 2 weeks (A), 3 months (B), 6 months (C), and 12 months (D). C = caudal, TF = transforaminal.

**FIGURE 2:**
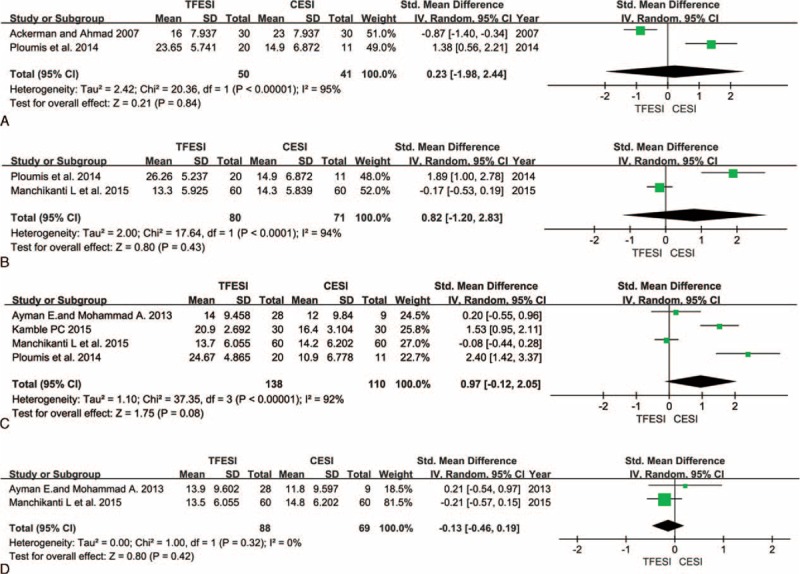
Comparison of TF versus C with respect to functional level after epidural steroid injections at 2 weeks (A), 3 months (B), 6 months (C), and 12 months (D). C = caudal, TF = transforaminal.

The data were collected and analyzed using Review Manager 5.3. Differences in pain and functional improvement between the TF and C groups were analyzed using the independent-samples *t* test under a random-effects model. The differences were displayed using a forest plot. The continuous data from the studies were reported as standardized mean differences (SMDs) with 95% confidence intervals (CIs). The I^2^ statistic^28^ (ranging from 0% to 100%) was applied to quantify between-study heterogeneity that was not attributed to chance (I^2^ = 0%–25%, no heterogeneity; I^2^ = 25%–50%, moderate heterogeneity; I^2^ = 50%–75%, large heterogeneity; and I^2^ = 75%–100%, extreme heterogeneity). Heterogeneity was interpreted via 8 statistics, which are used to analyze pain relief and functional improvement at 2 weeks, 3 months, 6 months, and 12 months, and the results are presented in forest plots (Figures 1 and 2). A *P* value <0.05 was considered statistically significant.

## RESULTS

### Literature Search

**FIGURE 3:**
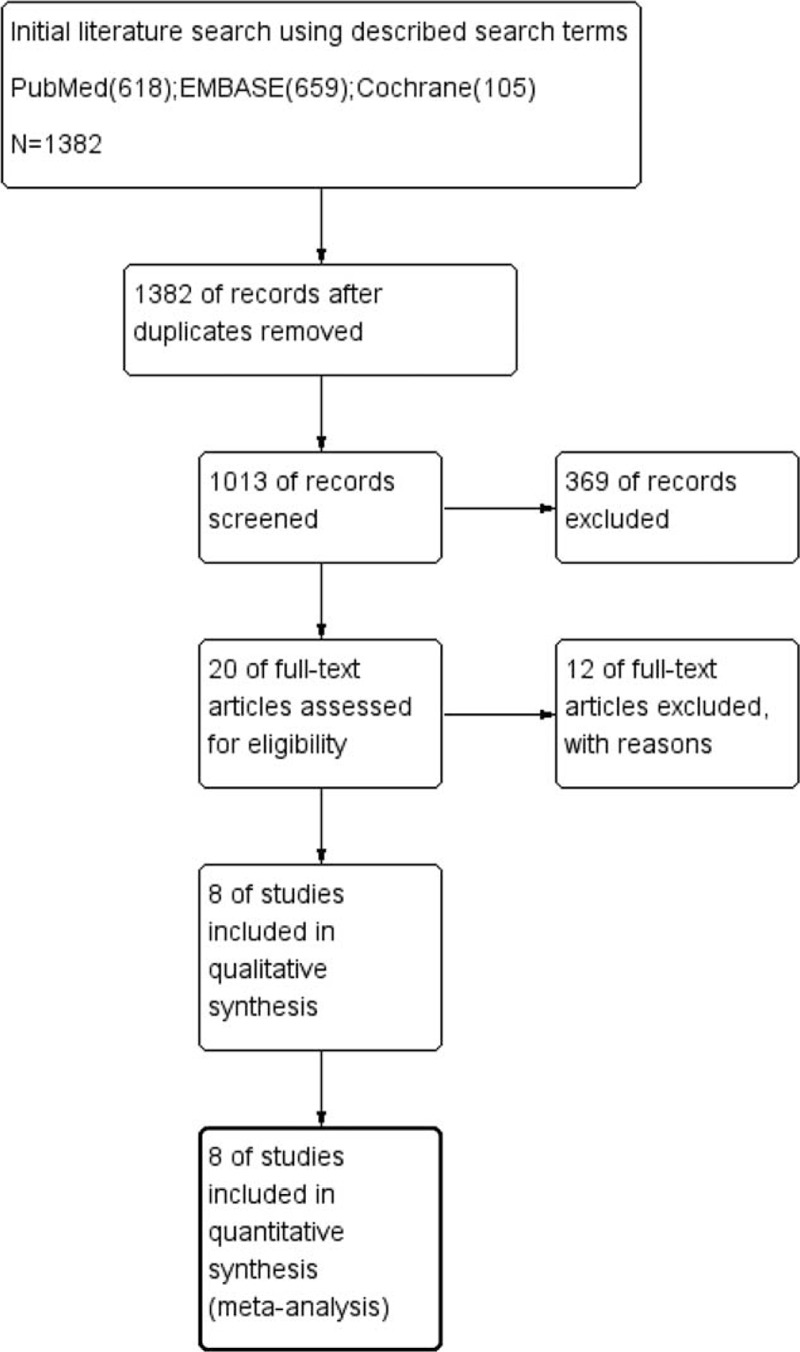
Flow diagram of the search and selection criteria for inclusion in this meta-analysis.

We identified 618 articles in PubMed, 659 articles in EMBASE, and 105 articles in the Cochrane Library. After the exclusion of 369 duplicate items, 1013 articles were screened for review, and 20 that met the inclusion criteria were selected. Twelve full-text articles were excluded due to either the absence of a comparison between TF-ESI and C-ESI or the absence of an appropriate statistical analysis. Eight studies^2,29–37^ were ultimately included in the meta-analysis (Figure 3).

### Demographics of the Studies Included in the Review

Six^2,30,32–34,37^ of the 8 studies were prospective studies, and two^29,31^ were retrospective studies. These studies included 664 patients, 278 of whom were included in the prospective studies and 386 of whom were included in the retrospective studies. Statistical analysis involving the 6 prospective studies was performed. Differences in age, sex, and injection level (Table 2) were noted; however, these differences were not statistically significant. Follow-up periods ranged from 2 weeks to 24 months in duration. Different types of corticosteroids were used in the studies, including triamcinolone, betamethasone, and depo-medrol (Table 3). The outcomes and clinical significance of the 6 prospective studies are summarized below and in Table 3, respectively.

### Risk of Bias in the Included Studies

**FIGURE 4:**
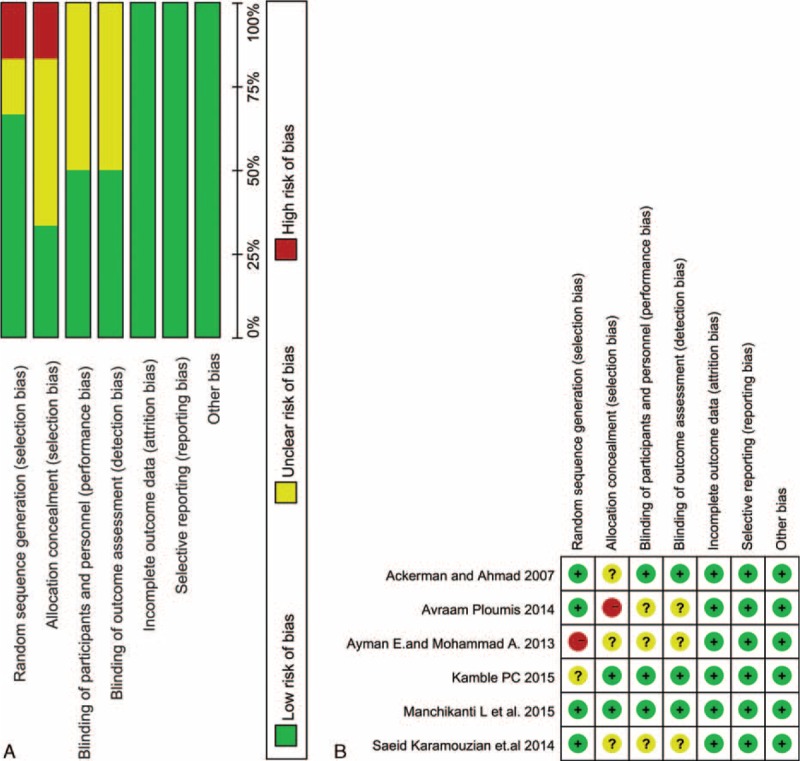
Risk of bias assessment of each included study. A, Risk of bias graph. B, Risk of bias summary.

We used the risk of bias tool implemented in Review Manager 5.3 to evaluate the risk of bias of the Cochrane Handbook for Systematic Reviews of Interventions. The particular information of the risk of bias of the included articles is demonstrated in Figure 4. Four^2,30,33–34^ of the 6 studies comprehensively described the generation of a randomized sequence, and the remaining studies^32,37^ did not demonstrate the randomization method. The patients were not blinded to treatment allocation in 1 study.^30^ It has 3 studies^2,34,37^ performed blinding, with the remaining studies being indistinct. All of the included studies displayed a low risk of bias for the incomplete outcomes, selective outcome reporting, and other biases.

### Change of Pain Level

All 6 prospective studies demonstrated reduced pain level, as indicated by changes in patients’ pain scores. Meta-analyses were performed at 2 weeks, 3 months, 6 months, and 12 months after the injections. Although the heterogeneity was high (I^2^ up to 93%), slightly better pain relief was observed in the TF groups at 2 weeks (Figure 1A), and no differences were noted after 3, 6, and 12 months of follow-up (Figure 1B–D).

Although their follow-up period was 24 weeks, Ackerman and Ahmad^2^ recorded patients’ pain scores for only 2 weeks in their prospective study. Ploumis et al^30^ followed their patients for 6 months and reported pain scores at 2 weeks, 3 months, and 6 months. The patients in the TF groups experienced superior pain relief at 2 weeks, although this improvement was not statistically significant.

Patients’ pain relief at 3 months was recorded in 2 studies.^30,34^ Ploumis et al^30^ observed slightly better pain relief after TF-ESI, although their findings were in contrast with those of Manchikanti et al.^34^ However, the differences between these studies were not significant.

Five prospective studies^30,32–34,37^ documented pain scores at 6 months. Three^30,32,37^ of the 5 studies demonstrated that the TF groups experienced significantly better pain relief than the C groups. The remaining study^33,34^ observed no difference between the 2 groups. Ackerman and Ahmad^2^ observed better pain relief in the TF group at 24 weeks. However, the pain scores in their prospective study were not recorded using either the VAS or NRS; their patients’ pain relief was graded as complete, partial, or no relief. In their study, 30 patients received C-ESI; 1 patient (3.3%) reported complete pain relief, whereas 16 patients (53.3%) reported partial pain relief and 13 patients (43.3%) reported no relief. Thirty patients received TF-ESIs; 9 patients (30%) reported complete pain relief, whereas 16 patients (53.3%) reported partial pain relief and 5 patients (16.7%) reported no relief.

Galhom and al-Shatouri^32^ and Manchikanti et al^34^ followed their patients for 12 months and provided data regarding pain relief. The pain level reported in these studies was 2.95 ± 1.759 versus2.60 ± 1.540 and 4.10 ± 1.391 versus4.30 ± 1.644 in the TF and C groups, respectively. No statistically significant differences in pain scores between the treatment groups were noted.

### Change of Functional Level

Five^2,30,32,34,37^ of the 6 prospective studies reported data on functional level rise. All 5 studies measured functional level using the ODI. Meta-analyses were performed at 2 weeks, 3 months, 6 months, and 12 months after the injections (Figure 2A–D).

Two studies^2,30^ reported data only for the 2-week time point, but noted significantly different outcomes after the injections. Ackerman and Ahmad^2^ observed that the C group experienced better functional improvement than the TF group, whereas Ploumis et al^30^ observed the opposite. Two prospective studies^30,32^ provided functional score data at 3 months after the injections, with differing results. Four studies^30,32,34,37^ recorded data at 6 months, and 2 studies^30,37^ observed slightly better functional improvement in the TF groups. No significant differences were noted among the groups after the injections were administered at the different time points.

### Complications

Few of the studies included in this meta-analysis recorded the occurrence of complications. Ackerman and Ahmad^2^ reported that no patients in their study presented with infection, headache, intravascular injection, and so on. In addition, no major complications were observed after administration of injections in the study conducted by Ploumis et al.^30^ Further, this group found that only 2 patients in the C group and none in the TF group required a second injection. Galhom and al-Shatouri^32^ reported that only 20% of the patients developed complications and that soreness at the injection site was the most common complication. Karamouzian et al^33^ found that only 1 patient suffered temporary paraparesis in the C group. Mendoza-Lattes et al^29^ did not observe the occurrence of complications; however, they reported that 10 of 39 patients required reinjection in the C group, in addition to 10 of 54 in the TF group.

## DISCUSSION

We summarized the results of studies comparing TF-ESI and C-ESI, and performed a meta-analysis to compare the effectiveness of TF and C routes for ESIs. We analyzed the effectiveness of these 2 approaches by evaluating improvements in patients’ pain and functional scores. We included 6 prospective studies and 2 retrospective studies involving 664 patients in our analysis. The TF approach seemed to facilitate slight improvements in pain relief within a short time period (no more than 6 months); however, no clinically or statistically significant differences in efficacy between TF-ESIs and C-ESIs were noted over longer periods of time. Five prospective studies measured patients’ functional improvement and observed superior results in the TF groups at both 3 and 6 months after treatment. However, no significant differences were observed between the 2 types of injections.

Several studies have indicated that ESIs improve patients’ pain and functionality.^18,24–27^ However, controversy exists regarding which route is the most beneficial and effective in the administration of epidural steroids. Previous studies and systematic reviews of ESIs have been hampered by their designs, baseline differences between the treatment groups, inadequate sample sizes, and an inability to confirm the location of the injection because fluoroscopy was not used.

Most experts believe that TF-ESI offers a more target-specific steroid delivery to the dorsal root ganglion and is therefore superior to C-ESI.^38,39^ However, C-ESI requires a high-volume injection (8–12 mL) to ensure that the epidural space is filled and that the drug is delivered to each vertebral level.^40^ By contrast, TF-ESI requires only a small volume (1–2 mL) and allows for the medication to be administered directly to the dorsal root ganglion.^41^

Several factors may clinically influence the outcomes of ESIs, thereby influencing the choice of the route of administration. With increasing age, the risk of developing radicular pain is higher. Older patients also tend to experience worse outcomes.^42^ Patients with a high disease burden^43^ and psychopathology,^44,45^ such as depression and other forms of psychological distress, may also have a worse outcome. Furthermore, a prolonged disease duration, lack of employment, smoking, the nature of a patients’ symptoms, and so on may affect the ESI results. And these may be the reasons why these included prospective studies generated different outcomes.

Most ESIs are used combining with local anesthetics. The local anesthetics are thought to have analgesic effects during the process of injection, which is beneficial for patients to relax. In these days, some researches^46–48^ show that ESIs combined with local anesthetics get a better effect on pain relief and functional level in managing chronic low back pain. Therefore, it is necessary to add the local anesthetic during the injections.

The 2 most common causes of complications of ESIs are related to inaccurate needle placement and medicine administration. Both types of injections may cause complications such as headache, soreness at the injection site, and toxicity.^49–53^ C-ESI is both the safest and the easiest epidural injection, and it does not always require fluoroscopic guidance. For the C route, there may be an increased risk of needle tip placement anterior to the sacrum or into the rectum, whereas TF-ESI carries an increased risk of trauma to the nerve root during needle placement, which may result in paraplegia in rare instances.^54^ Further, all types of steroids may cause adverse events and complications, such as osteoporosis, necrosis of bone, fluid retention, steroid myopathy, weight gain, and so on. Therefore, clinicians should pay attention and use caution when performing ESIs.

Although no significant differences were noted with respect to either pain or functional improvement, TF-ESI was slightly more effective with respect to radicular pain management over a short duration (no more than 6 months). However, over 12 months (a longer time period), C-ESIs exhibited a slightly better impact on both pain and functionality. Ackerman and Ahmad^2^ documented both change of pain score and functional score after only 2 weeks, although their patients were followed for 24 weeks. Ploumis et al^30^ also performed a randomized, blinded, prospective trial, and recorded changes in patients’ pain and functionality between 2 weeks and 6 months after treatment. These studies observed similar outcomes in degree of pain relief, but contrasting results in functional level at 2 weeks (Figures 1A and 2A). Said discrepancy was attributed to differences in patients’ ages; the average age of the patients in the study by Ackerman and Ahmad was 34.00 ± 5.00 years in the TF group and 36.40 ± 4.00 years in the C group, whereas the average age of the patients in the study by Ploumis et al was 64.70 ± 8.05 years in the TF group and 67.20 ± 9.45 years in the C group. The types of medication used were also different and included either triamcinolone or betamethasone. When we analyzed the improvements in both pain and function at 3, 6, and 12 months (Figures 1B–D and 2B–D) after the ESIs, we observed that the TF group experienced slightly less improvement than the C group in the study by Manchikanti et al,^34–36^ which was in contrast to the results of other studies. The primary reason for this discrepancy is because the study by Manchikanti et al summarized the results of 3 separate RCTs, and although these 3 studies had the same design, they were performed at different times.

One limitation of this study was its small sample size, which made assessing the effectiveness of the interventions on different patient outcomes difficult. The small sample size may be attributed to the inclusion and exclusion criteria utilized in this review, as the studies that were ultimately included in this systematic review did utilize consistent inclusion and exclusion criteria. Another limitation of this review was that corticosteroid dosages differed among the included studies; in some cases, the dosages differed between groups enrolled in the same study. Third, many of the studies lacked data regarding complications, additional injections, the need for local anesthesia, and the need for fluoroscopic guidance.

## CONCLUSIONS

Most clinicians believe that TF-ESI exhibits superior efficacy and is being performed with increasing frequency, although this belief seems to be unsubstantiated.^23,38,39,55^ Our study has demonstrated that TF-ESI and C-ESI are similarly effective in managing lumbosacral radicular pain. Before selecting a steroid injection route for the management of radicular pain, the benefits and risks of the approaches discussed herein must be taken into consideration, as the outcomes of this review were not significantly different between the 2 treatment groups. Additional studies must therefore be performed to guide clinical decision-making.
